# Open Platform Cameras Based Bio-Imaging Evaluation System†

**DOI:** 10.3390/s21206727

**Published:** 2021-10-10

**Authors:** Ji-Yeon Baek, Jong-Dae Kim, Yu-Seop Kim, Chan-Young Park, Ji-Soo Hwang

**Affiliations:** 1 Help-Me Law Firm, Seoul 06158, Korea; qorwldus0310@naver.com; 2 School of Software, Hallym University, Chuncheon-si 24252, Korea; kimjd@hallym.ac.kr (J.-D.K.); yskim01@hallym.ac.kr (Y.-S.K.); 3 Bio-IT Research Center, Hallym University, Chuncheon-si 24252, Korea

**Keywords:** gel-document system, gel electrophoresis, bio-imaging, open platform-based camera

## Abstract

With the active development of mobile devices, a variety of ultra-small, high-definition, and open platform-based cameras are being mass-produced. In this paper, we established an emulation system to verify the bio-imaging performance of the bulky and expensive high-performance cameras and various smartphone cameras that have been used in bio-imaging devices. In the proposed system, the linearity of the brightness gradient change of four types of cameras was compared and analyzed. Based on these results, three cameras were selected in order of excellent linearity, and gel image analysis results were compared.

## 1. Introduction

Genetic information is contained in DNA or RNA. Analysis technology using a very small amount of genome has been applied in various fields [1,2,3,4]. In particular, it is obtained using polymerase chain reaction (PCR), a molecular biological technique that amplifies trace genes [5,6,7]. Then, in order to confirm whether the product is amplified and to analyze genetic information, it performs an electrophoresis process to analyze nucleic acids or proteins. The gel-document system is a device that enables image analysis by photographing agarose gel after electrophoresis, in order to analyze the nucleotide sequence or size of a gene by analyzing the image obtained from electrophoresis [8,9,10,11,12,13,14].

As for the optical sensor used in commercial gel-document systems, the larger the image sensor the higher the image quality, so expensive high-performance CCD cameras (portable gel-document system, manufacturer: DAIHAN, USD 2728) or DSLR (digital single lens reflex) cameras using CMOS sensors (Felix 2010-R, manufacturer: Biostep^®^, EUR 3445) are mainly used. However, these sensors are not only expensive but also large in size. In the case of a DSLR camera, it is difficult to miniaturize the size of the entire gel-document system, because the focal length must be maintained to at least 30 cm.

With the development of mobile devices, it has become easier to obtain a camera based on an ultra-compact, high-performance open platform at an affordable price. These cameras have the advantage of being small in size and have a short focal length, making it possible to implement a gel-document system with excellent specifications and cost-effectiveness [15,16,17].

This paper selects a camera with excellent linearity through an emulation experiment that compares the linearity of inclination according to the brightness of three types of open platform-based cameras, with different prices and sizes based on the brightness of DSLR cameras mainly used in commercial devices [18].

In this paper, a Canon EOS 1100D was selected as a representative DSLR camera used in commercial equipment, and a Sony IMX179, AR0130, and PICAM were selected among cameras of various sizes and prices under USD 60 as open platform-based cameras (price excluding individual lenses).

The Sony IMX179 and AR0130 were selected as open platform-based cameras with excellent linearity by conducting comparative emulation experiments on the linearity of three types of open platform-based cameras, based on the brightness of DSLR cameras.

In order to compare the system sensitivity of commercial equipment with the three types of cameras selected through emulation experiments, band volumes of images after electrophoresis of agarose gel taken with these cameras were compared and analyzed [19,20,21,22].

As a result of analyzing the images, it was found that the DSLR camera has the best maximum gain. However, if the standard maximum gain is adjusted to the maximum gain of the AR0130, the gain of the DSLR camera was similar to that of the AR0130. Therefore, even if the DSLR camera is replaced by the AR0130, it is expected to show sufficient performance, which shows that it is possible to implement a low-cost, small-sized gel-document system even with an open platform-based camera.

## 2. Materials & Methods

### 2.1. Gel-Document Emulation System

**Figure 1:**
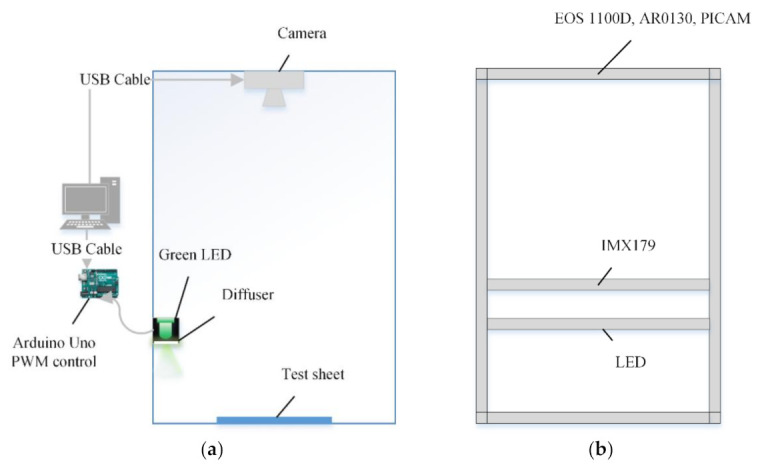
Gel-document emulation system: (**a**) configuration; (**b**) fixed position.

Figure 1 shows the configuration of the gel-document emulation system. The microcontroller for camera and lighting control is connected to the PC with a USB cable. A green LED is used for lighting and PWM (pulse width modulation) is controlled through a microcontroller to change the brightness.

There are 4 types of cameras used and Table 1 shows the information on the cameras used in the experiment. The DSLR camera used in the comparative experiment is one of the image devices used in actual commercial equipment. Among them, the comparison experiment using the Canon EOS 1100D was conducted considering that it could be said to be a comparison experiment with commercial equipment. Moreover, open platform-based cameras were selected from products under USD 60. The Sony IMX179 (8MP autofocus, USD 36) is used as an optical sensor in DuxGelDoc. AR0130 (1.3 MP, USD 35) is a camera that additionally includes a CS-mount varifocal 2.8–12 mm lens and can adjust brightness, contrast, color, saturation, gamma, definition, and white balance. For PICAM, Sony IMX 477R stacked back-illuminated sensor is used and has a resolution of 12.3 MP. Moreover, this camera uses an 8–50 mm zoom lens.

Figure 1b shows the fixed positions of the green LED and cameras in the emulator. The height of each camera in the emulation system was fixed at a position where the ratio of the width was 80% when the test sheet was shot with each camera. Except for the IMX179 camera, three types of cameras were fixed 300 mm above the test paper, the IMX179 camera was fixed at 110 mm from the reference position, and the green LED was fixed at 75 mm. To compare the brightness of each camera, the brightness of the optical sensor IMX179 installed in DuxGelDoc (manufacturer: Biomedux Co., Ltd., USD 1348), a gel-document system jointly developed with the affiliated research team, was compared as the reference. The maximum exposure of this sensor was 1 second, and the slope of PWM100 at the maximum exposure was calculated. Based on this, the exposure time of the remaining cameras was adjusted. (The Arduino Uno provides an 8-bit PWM output, but in this experiment the brightness is based on PWM 100.) Table 2 shows the slope and exposure (sec) when PWM is 100 for each camera [17,23,24].

**Figure 2:**
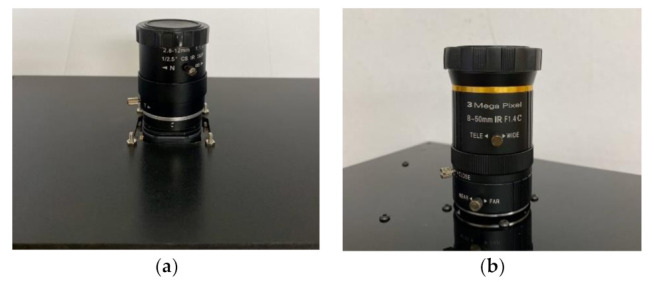
Lens aperture: (**a**) AR0130; (**b**) PICAM.

Figure 2 shows the lenses used for AR0130 and PICAM. These lenses used low-cost lenses, and since the aperture adjustment value was not marked on these lenses, it was impossible to set the exact aperture value. However, the value of the aperture was taken by adjusting the slope of the brightness in PWM 100 of each camera, to be close to that of the IMX179.

**Figure 3:**
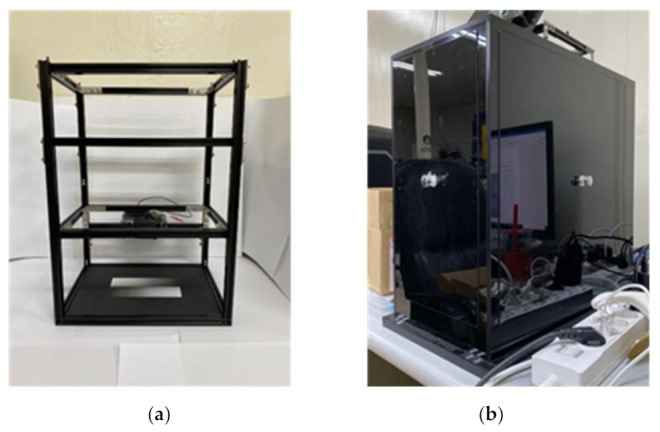
Gel-document emulation system: (**a**) emulator; (**b**) dark room.

Figure 3a shows the emulator produced by this research team. As shown in Figure 2b, the experiment was conducted in a dark room made of black acrylic with a size sufficient for installing the emulator. In order to set the light brightness similar to DuxGelDoc’s light, the light part consisting of a green LED (product number: C503B-GAS-CB0F0792CT-ND, Digikey, Thief River Falls, MN, USA), and a diffuser (3M 3635-70, 2set) was adjusted by changing the PWM signal from the microcontroller (Arduino Uno, pin #9 (490Hz)).

#### 2.1.1. Analysis Method 1

In this method, before proceeding with the comparative experiment, two experiments were performed to confirm the accuracy of the comparative method. In the first experiment, it was analyzed whether the reflectance was constant regardless of the brightness of the light. In the second experiment, we analyzed whether the most accurate experimental results can be derived by fitting the image profile with an equation of what order. In this process, the following four equations (Equations (1)–(4)) were used:

(1) $$y_{\omega }(t):0\;\leq \;t\;\leq \;1,$$

(2) $$p_{\omega }\;=\;polyfit(t,y_{\omega },n),$$

(3) $${\hat{y}}_{\omega }(t)\;=\;polyval\left(p_{\omega },t\right)$$

(4) $$e_{w}\;=\;np.\mathrm{var}\left(y_{w}\;-\;{\hat{y}}_{w}\right)$$

Before proceeding with the two experiments, to match the pixel units of each camera, the pixel positions were normalized to 0~1 in the profile of the image, with PWM = ω through Equation (1). Equation (2) was used to obtain polynomial coefficients by performing nth-order polynomial fitting. Equation (3) was derived from the polynomial value of the function value of the parameter obtained in Equation (2).

To verify the first experiment, a linear plot was drawn by multiplying 100 elements between 0 and 1 by the value of the function derived from Equation (3) by each image brightness (PWM = (100, 80, 60, 40, 20)) using the linspace function. For comparison, the function made by multiplying the PWM 100 parameters for each camera by constant 0.8, 0.6, 0.4, and 0.2 was multiplied by 10 elements between 0 and 1 using the linspace function and displayed as a dotted line on the linear plot. At this time, the PWM 100 image was taken under the brightest lighting condition, so the PWM 100 parameter was taken as the standard.

In the second experiment, the slope change according to light brightness was verified by plotting PWM vs. slope for the slope of the function obtained by Equation (3). With Equation (4), the errors in the first to sixth-order equations of each camera were calculated.

To compare the linearity of the errors obtained in this method, PWM vs. error for each order of the cameras used is plotted. At this time, all cameras shot in the order PWM = (100, 80, 60, 40, 20).

#### 2.1.2. Analysis Method 2

In the second method, an actual comparison of four cameras was performed. Through the polynomial parameters obtained by fitting the PWM 100 image taken under the brightest lighting for each camera, we found the constant value with the smallest error for PWM 80 to 20 and compared the error at that time.

The parameter value of PWM 100 was obtained by Equation (5), and the function value and error for it were calculated using Equations (6) and (7).

(5) $$p_{100}\;=\;polyfit\left(t,y_{100},n\right)$$

(6) $${\hat{y}}_{100}(t)\;=\;polyval\left(p_{100},t\right)$$

(7) $$e_{100}\;=\;np.\mathrm{var}\left(y_{100}\;-\;{\hat{y}}_{100}\right)$$

For PWM = (80, 60, 40, 20), the following three equations (Equations (8)–(10)) were used to obtain the constant that minimizes the error.

The linearity of the constant obtained through the equation could be verified through the PWM vs. constant for the third-order parameter of each camera. When multiplied by the constant *d*, which minimizes the error, the result of the error value is shown through the PWM vs. error plot. At this time, the constant *d* = (1, 0.8, 0.6, 0.4, 0.2) baseline was also drawn so that it could be recognized at a glance.

(8) $$p_{w}\;=\;d\;\times \;p_{100}$$

(9) $${\hat{y}}_{w}(t)\;=\;polyval\left(p_{w},t\right)$$

(10) $$e_{w}\;=\;np.\mathrm{var}\left(y_{w}\;-\;{\hat{y}}_{w}\right)$$

### 2.2. Gel-Document System Experiment

**Figure 4:**
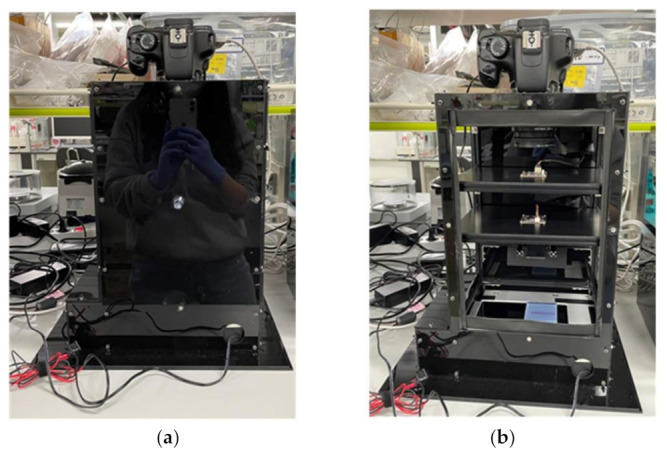
Low-cost gel-document system: (**a**) external appearance; (**b**) internal structure.

Figure 4a is a low-cost gel-document system manufactured by our research team to take pictures using multiple cameras, and Figure 4b shows the inside of the gel-document system. This system was manufactured by adding a UV transilluminator (manufacturer: MAESTROGEN, product name: UV Transilluminator_UUV-01 UltraSlim, viewing size: 8×15 cm, UV lamp: T5-6W-301 1pc, filter: UltraSafe UV blocking) to the gel-document emulation system created in the previous emulator experiment, and the exterior was made of black acrylic to create a dark room.

The DSLR camera (EOS1100D, Canon), IMX179 used in the existing gel-document system, and the AR0130 selected through emulator experiments were taken with an emission filter installed. The emission filter used in the experiment was Tiffen’s Orange Filter (manufacturer: Tiffen, product name: Tiffen 58 mm 21 Filter (Orange), size: 58 mm, photo filter effect type: ultraviolet).

A PCR test was performed to compare the performance of the selected camera, and nuclease-free water (Qiagen) was added to the amplified CT (chlamydia trachomatis) DNA sample and diluted to a concentration of 1, 1/2, 1/5, 1/10, 1/20. Five types of samples with different concentrations were injected into an agarose gel (0.5x TBE) and subjected to electrophoresis for 25 min. The agarose gel was photographed with three types of cameras selected in the previous emulator experiment, and these images were analyzed. The shooting conditions of each camera were set, as shown in Table 3, based on the previous emulator test results. At this time, to proceed with shooting with the same brightness, the image was taken 120 seconds after turning on the UV transilluminator.

As can be seen from the image size in Table 3, the image pixel units of the cameras used are all different. For an accurate comparison, the OpenCV functions cv2.getPerspectiveTransform() and cv2.warpPerspective() were used to match the images of the agarose gel taken in different pixel units, and then the band images to be used for analysis were output for each experiment. Since the brightness of the agarose gel may decrease as the exposure time to UV increases, two types of cameras were taken alternately for comparison.

In other words, in a comparative experiment using a DSLR camera and AR0130, two gels were used to shoot. First, the first gel was photographed in the order of DSLR→AR0130→DSLR (Experiment 1), and the second gel was photographed in the order of AR0130→DSLR→AR0130 (Experiment 2), and then the band volumes of the images were compared. A comparison experiment between AR0130 and IMX179 was conducted in the same way (AR0130→IMX179→AR0130 (Experiment 3), IMX179→AR0130→IMX179 (Experiment 4)).

**Figure 5:**
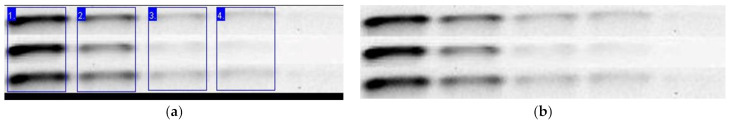
Gel image analysis: (**a**) band image; (**b**) exp #1 band image.

Each band image was used to compare camera performance through band volumes analyzed using a gel analyzer, as shown in Figure 5.

Comparison of camera performance was carried out by obtaining the coefficient of determination for the band volume and comparing the ranks, comparing the R^2^ average of the primary and tertiary images and the R^2^ value of the secondary image for each experiment, and comparing the slope.

At this time, since AR0130 and IMX179 were set as the maximum exposure value, brightness comparison was also analyzed.

## 3. Experimental Results

### 3.1. Emulation Test Results

#### 3.1.1. Analysis Method 1

**Figure 6:**
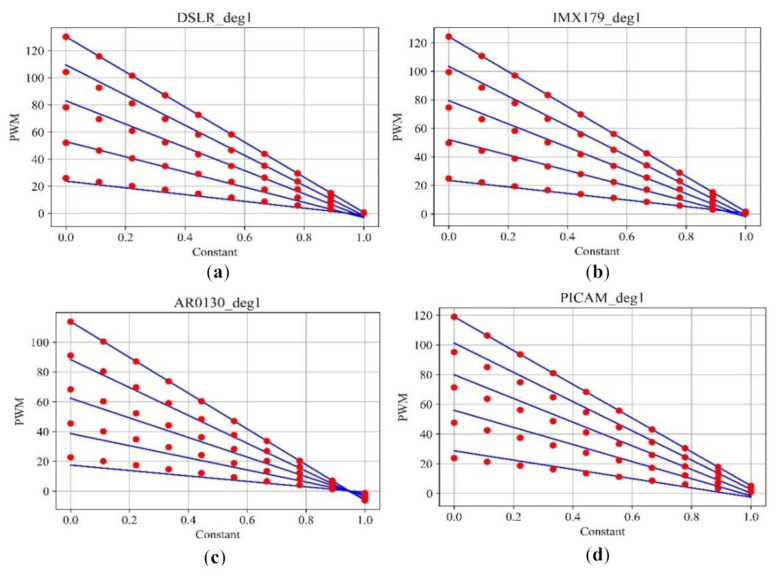
Relationship diagram for the first-order parameters for each camera: (**a**) DSLR; (**b**) IMX179; (**c**) AR0130; (**d**) PICAM.

Figure 6 is a plot drawn to confirm the first experimental condition of the first method. The *x*-axis is the range of integers (0 to 1) multiplied using the linspace function, and the *y*-axis represents the PWM brightness. As can be seen in Figure 6, it was confirmed that the blue line drawn using the function generated by the parameters for each image brightness in the four cameras photographed matches the red dot drawn by the function created by multiplying the PWM100 parameter by the constants 0.8, 0.6, 0.4, and 0.2. This shows that the reflectivity of each camera is constant regardless of the brightness of the light. Rather than comparing the red and blue lines for exact matches in the analysis in Figure 6, we wanted to see how linear the red points are with respect to the blue line. Moreover, since Method 2 compares the linearity for each camera, it cannot be seen that these results affect the primary interpretation of the results.

**Figure 7:**
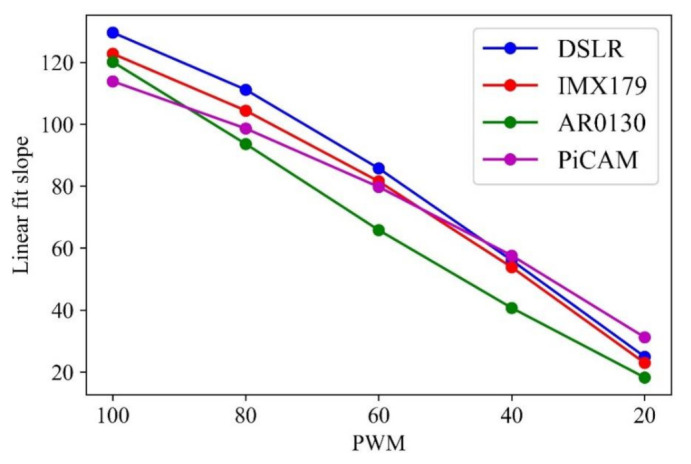
First-order parameter slope according to PWM for each camera.

In Figure 7, which plots the results of the second experiment, it can be confirmed that the change in slope according to the brightness of all cameras is proportional. It can be said that the better the camera performance is, the more linear the lighting brightness and the slope obtained through it. Among the four cameras, the slope of the AR0130 was the most linear.

**Figure 8:**
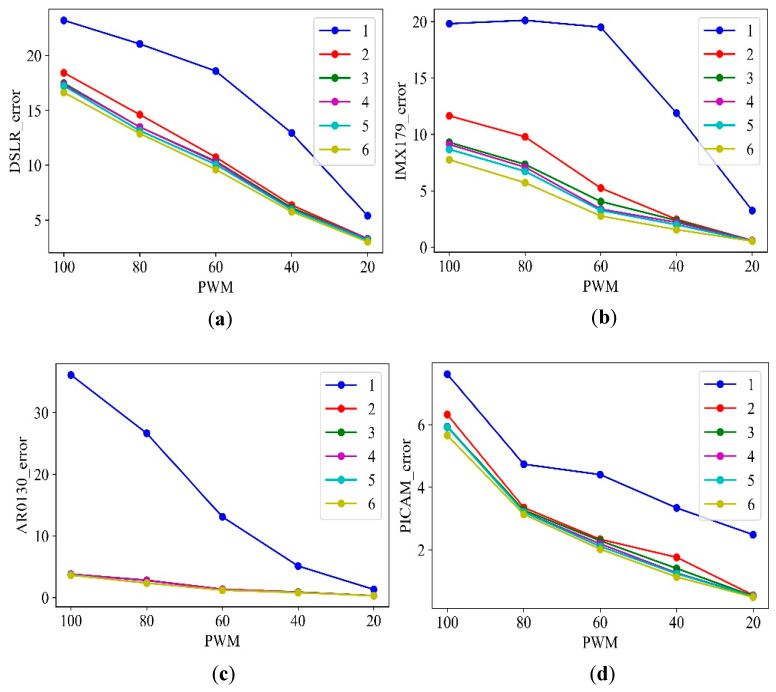
Errors according to PWM for each order of cameras: (**a**) DSLR; (**b**) IMX179; (**c**) AR0130; (**d**) PICAM.

Figure 8 shows the relationship between PWM and error for each order of each camera. In this experiment, since the lighting was not uniform, it was difficult to conclude only with the first-order equation, so the analysis was performed while increasing the order. As a result of comparing the errors from the first-order to the sixth-order equation, it was found that there was no significant difference when the errors of all cameras were third-order or higher. In other words, the large or small error is not a problem in this experimental result. What we want to find out in Figure 8 is from which order equation is there no change in error. As shown in Figure 8, it can be seen that sufficient results can be obtained even with the third-order equation, since similar results are shown from the results of the third-order equation or higher.

#### 3.1.2. Analysis Method 2

**Figure 9:**
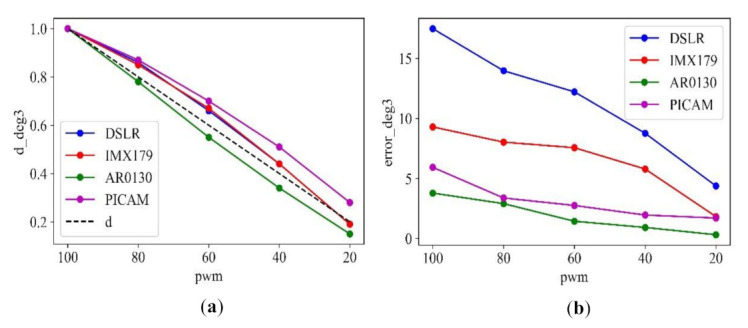
Comparison of the 3rd parameter for PWM 100 to 20 of each camera: (**a**) constant; (**b**) error.

Based on the results of the second experiment in Method 1, in Method 2 the constants and errors were compared based on the third-order parameter. As a result of verifying the camera that is most similar to the constant baseline, e.g., the most linear, through Figure 9a, which plots the results of Method 2, it was found that the AR0130 results were the most linear among the four cameras. Based on the results of Method 1 and Method 2, the AR0130, with good linearity, performed the best out of the four cameras. Therefore, the AR0130 was selected as the camera to be used in the agarose gel image analysis experiment after the actual electrophoresis test.

### 3.2. Gel-Document System Analysis Results

Table 4 below shows the ranking comparison results for values when comparing the band volume of each camera in the four experiments. In a comparison experiment between the DSLR and AR0130, the AR0130 showed better performance than the DSLR, and in a comparison experiment between the AR0130 and IMX179, it was found that the AR0130 performed better. In the result of comparison between the DSLR camera and AR0130, the performance of the DSLR camera is determined by lens performance, CMOS image sensor pixel count and performance, and image processing engine performance. Compared to the open platform cameras, the image processing engine performance, which can influence the performance, is superior, so it can be said that the sensitivity or linearity is excellent.

**Figure 10:**
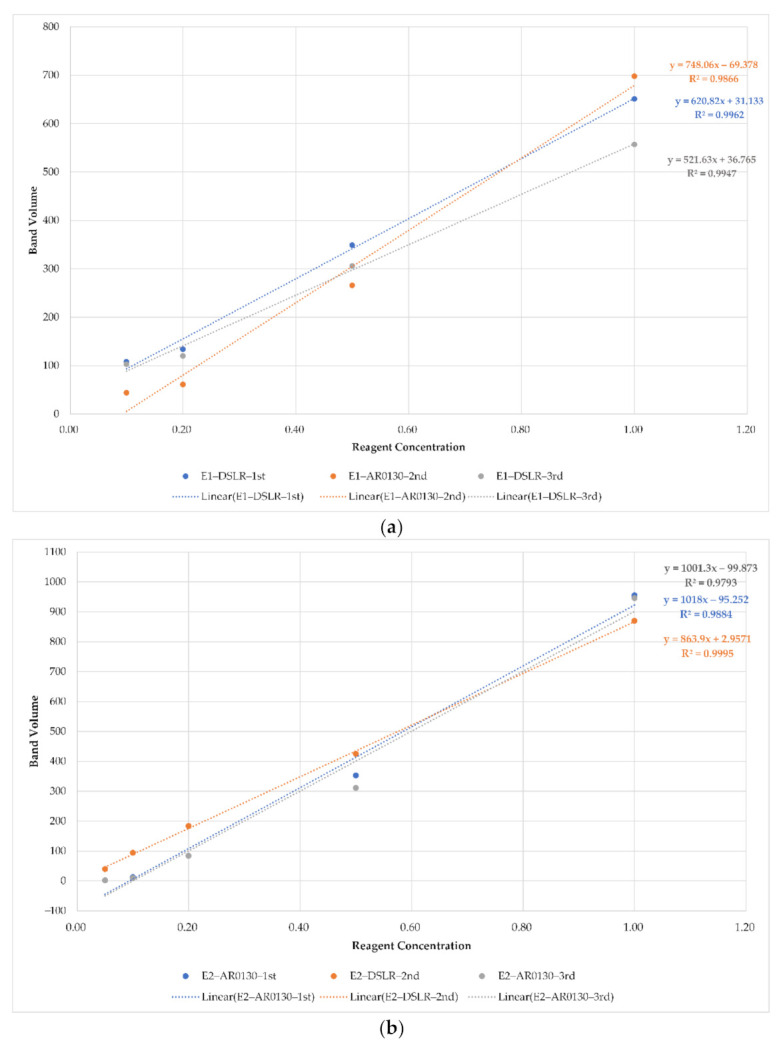

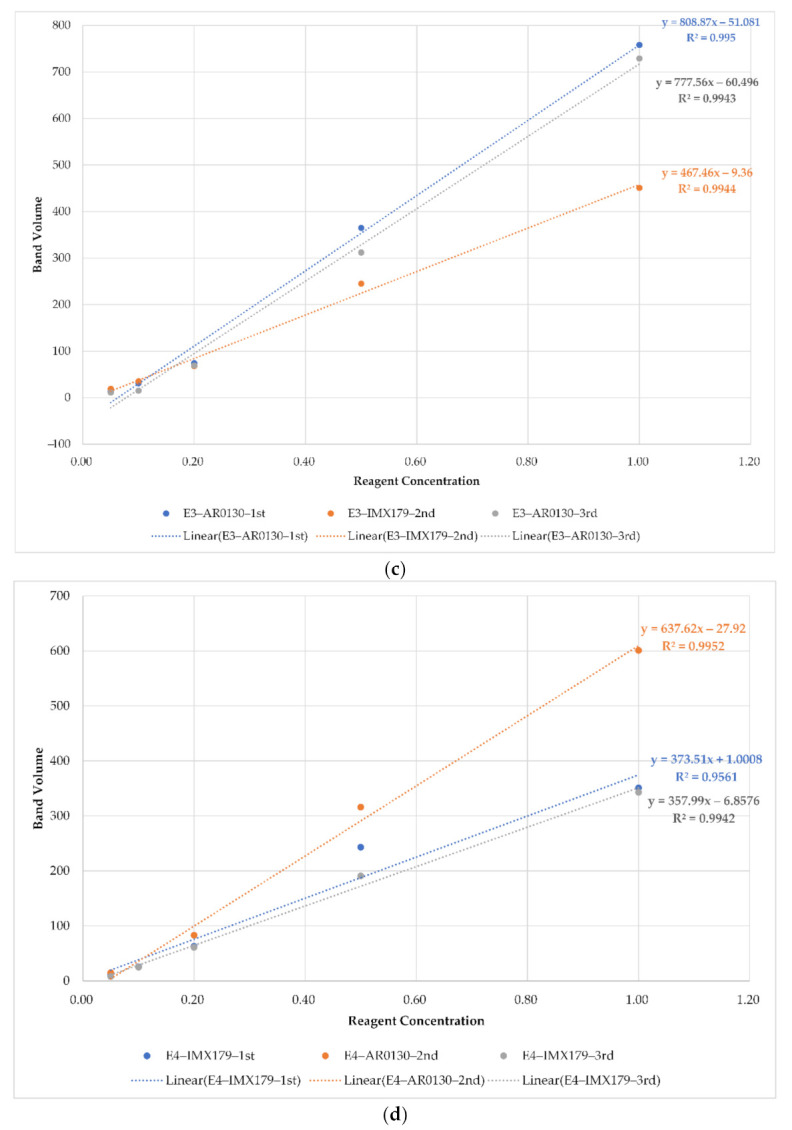
Band image analysis: (**a**) exp #1; (**b**) exp #2; (**c**) exp #3; (**d**) exp #4.

Figure 10 shows the results of comparing the average of the band volumes of the first and third images for each experiment with the second image. It was confirmed that the linearity of the DSLR and AR0130 was similar, and there was no significant difference between the two experiments when the slopes were compared. This shows that the exposure of the reference camera, DSLR, is set similar to the maximum exposure of the AR0130.

In the comparison between the AR0130 and IMX179, the linearity of the former was better, and it was found that the comparison of the slope also better expressed the difference in the concentration of the band volume in the former.

The purpose of examining the brightness by changing the PWM is to investigate the system sensitivity according to the change of lighting. Sensitivity can be expressed by $\frac{\Delta Brightness}{\Delta PWM}$. If this result is constant, then the trend in the result should be linear.

If the slope is large, the band brightness change should be linear. In other words, if the slope is steep, the sensitivity is high. In addition, the linearity was investigated by obtaining the coefficient of determination R^2^ for the gel image, and the system sensitivity was analyzed through this. It can be seen that the closer R^2^ is to one, the better the linearity, e.g., the sensitivity. Signal-to-noise ratio (SNR) is expressed as $\mathrm{SNR}\;=\;\frac{R^{2}}{1\;-\;R^{2}}$, and the coefficient of determination R^2^ is expressed as $R^{2}\;=\;\frac{SNR}{1\;+\;SNR}$. Therefore, as R^2^ increases, SNR also increases, so it can be seen that the system sensitivity and SNR have a proportional relationship.

## 4. Conclusions & Discussion

With the development of open platform-based cameras, ultra-compact, low-cost, high-performance cameras are easily accessible. If the price and size can be reduced by applying these to the gel-document system, the gel-document system, which was not easily accessible, can be generalized.

In this paper, the IMX179 and AR0130 were selected by comparing the linearity of the brightness of three types of open platform-based cameras. When comparing camera performance through gel analyzer analysis, the AR0130 showed better performance between the open platform-based cameras AR0130 and IMX179.

Based on this result, it is possible to replace the IMX179 installed in the system developed by this research team with the AR0130. In addition, in the experiment comparing the DSLR camera and the AR0130, it can be seen that the performance of the DSLR camera is superior to that of the AR0130, but there is no significant difference when compared numerically. This means that the DSLR camera can be replaced by the AR0130, enabling the implementation of a low-cost, compact gel-document system using an open platform-based camera.

It is difficult to definitively claim which camera is better through the gel-document experiment, since it cannot be said which is more reasonable between the ranking test and the average comparison method used in the experimental analysis process. This requires the process of comparing and analyzing the same experiment 10 times or more.

In addition, in the plot graph drawn to compare the linearity of each camera, the AR0130 decreases linearly according to brightness, but the DSLR camera and IMX179 descend by drawing a curve. The same result could be seen in the comparison of the slope according to the change in PWM brightness conducted in the emulator experiment. This shows that there is no problem in selecting a camera based on the emulation test result alone. In future experiments, it shows that performance comparison is possible through the evaluation of agarose gel images taken with a camera and a DSLR camera, selected based on the results presented above.

The more linear the camera is in bright places, the better. The AR0130 showed better linearity in bright places. Therefore, it is also meaningful to analyze darkly photographed images. Since it is more advantageous if it is linear in brightness that can be distinguished by human eyes, it is expected that better results will be obtained if additional experiments are conducted to perform rank comparison and average analysis only with bright parts.

## Figures and Tables

**Table 1:** Camera information.

	DSLR	IMX179	AR0130	PICAM
Model No.	EOS 1100D	HBVCAM - 8M1822 V11	KYT-U130-CS2812	Raspberry pi High Quality Camera
Lens	EF-S 18–55 mm f/3.5–5.6 IS STM	Supplied lens (lens:5P)	Supplied lens (CS mount varifocal, 2.8~12 mm lens)	8–50 zoom lens (C to CS mount used)
Sensor feature	12MP	8MP	1.3MP	12.3MP
S/W	DSLR remote pro	myCAM	myCAM	Raspberry pi4 (connect to PHP server)
Price (USD)	403.96	36	35	56.44 (Body) (Lens: 47.6)

**Table 2:** Camera shot initial conditions.

	PWM 100 Linearity	Exposure (Sec)	Shooting Condition
DSLR	126	1/8 (TV)	ISO 1600, AV:5.0
IMX179	122	1	Focus: Autofocus White balance: Auto
AR0130	120	0.5	Focus: Autofocus White balance: Auto
PICAM	113	1	Exposure mode: off

**Table 3:** Camera shot conditions.

	Shooting Height	Exposure (Sec)	Image Size (Pixel)	Aspect Ratio (%)
EOS1100D	300	1/5, 1/6, 1/8, 1/10	4272 × 2848	37
AR0130	200	1/2, 1/4, 1/8, 1/16	1280 × 960	33
IMX179	133	1, 1/2, 1/4, 1/8	3264 × 2448	30

**Table 4:** Results by experiments.

Exp #1	DSLR 1st shot	AR0130 2nd shot	DSLR 3rd shot
R^2^	0.9962	0.9866	0.9947
Rank	1	3	2
**Exp #2**	**AR0130 1st shot**	**DSLR 2nd shot**	**AR0130 3rd shot**
R^2^	0.9884	0.9995	0.9793
Rank	2	1	3
**Exp #3**	**AR0130 1st shot**	**IMX179 2nd shot**	**AR0130 3rd shot**
R^2^	0.995	0.9944	0.9943
Rank	1	2	3
**Exp #4**	**IMX179 1st shot**	**AR0130 2nd shot**	**IMX179 3rd shot**
R^2^	0.9561	0.9952	0.9942
Rank	3	1	2

## Data Availability

Data sharing not applicable.
